# A geometric method for contour extraction of Drosophila embryos

**DOI:** 10.1186/s12918-017-0478-1

**Published:** 2017-12-14

**Authors:** Qi Li, Yongyi Gong

**Affiliations:** 1 Western Kentucky University, 1906 College Blvd., Bowling Green, 42101 KY USA; 2 Cisco School of Informatics, Guangdong University of Foreign Studies, Guangzhou, 510006 People’s Republic of China

**Keywords:** Drosophila embryo, Contour extraction, Concave shape, Dominant point, Geometric sequence

## Abstract

**Background:**

High resolution images of Drosophila embryos in their developmental stages contain rich spatial and temporal information of gene expression. Automatic extraction of the contour of an embryo of interest in an embryonic image is a critical step of a computational system used to discover gene-gene interaction on Drosophila.

**Results:**

We propose a geometric method for contour extraction of Drosophila embryos. The key of the proposed geometric method is k-dominant point extraction that is a generalization of 3-dominant point extraction proposed in our previous work. Based on k-dominant point extraction, we can approximate a connected component of edge pixels by a polygon that can be either convex or concave. The test on BDGP data shows that the proposed method outputforms two existing methods designed for contour extraction of Drosophila embryos.

**Conclusions:**

The main advantage of the proposed geometric method in the context of contour extraction of Drosophila embryos is its ability of segmenting embryos touching each other. The proposed geometric method can also be applied to applications relevant to contour extraction.

## Background

High resolution images of Drosophila embryos in their developmental stages contain rich spatial and temporal information of gene expression. They have become a valuable instrument for micro-biologists to discover gene-gene interaction [1]. Automatic extraction of the contour of an interest embryo in an image is a critical step of a computational system for the discovery of gene-gene interaction on Drosophila [2].

In general, Drosophila embryonic images contain substantial amount of variations [3, 4]: i) imaging conditions, such as contrasts, scale, orientation, and neighboring embryos, ii) gene expression patterns, and iii) developmental stages. Most existing methods were developed upon low-level image features, such as edge pixels or pixels with a high deviation of grayvalues in a local window [3–10]. Peng and Myers [5] proposed a method that uses the standard deviation of the local windows of a pixel to classify the pixel as a foreground or background pixel. Their method applies a 8-neighbor-connectivity region-growing method to extract the contour of an embryo. Pan et al. [6] applied a variant of Marquardt-Levenberg algorithm to estimate an optimal affine transformation to register localized embryos into an ellipsoidal region. Puniyani et al. [7] proposed an edge detection based method that assumes a number of heuristic constraints, including object size, convexity, shape features (e.g., ratio of the major over minor axis of an object), and the percentage of overlapping regions. Frise et al. [8] developed the method of Peng and Myers [5] by adding three morphological operations on a binary image: i) removal of isolated pixels, ii) dilation, and iii) majority processing. Futhermore, Frise et al. [8] proposed a heuristic algorithm to separate the embryo of interest from multiple touching embryos, with the assumption that the center of the embryo of interest is the image center. Mace et al. [9] proposed an eigen-embryo method to extract the contour of embryos, where a particle swarm optimizer was used to reduce the computational cost of searching optimal eigen parameters. Li and Kambhamettu [3] proposed a quadratic curve model to initialize the contour of the embryo of interest based on edge pixels, and applied an active contour model to refine embryo contours. Bessinger et al. [10] proposed criteria to select the optimal connected component of edge pixels in the scale space of an input image. Li [4] proposed algorithms to detect and restore deficiencies and faults of primal sketch tokens that occur when a targeting object is surrounded by a complex background.

**Fig. 1:**
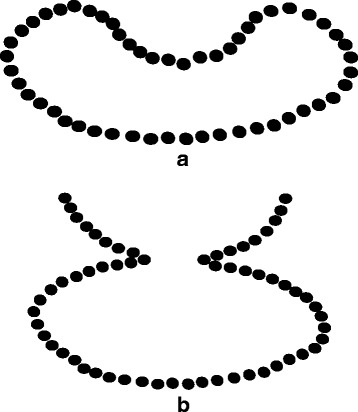
Two circumstances for the introduction of k-dominant points. **a** the contour of a Drosophila embryo forms a concave shape, and **b** the contour of two Drosophila embryos that touch to each other forms a concave shape

In this paper, we propose a geometric method for contour extraction of Drosophila embryos, and the key of the proposed method is k-dominant point extraction. In the context of rectangular shape detection [11], we have proposed 3-dominant point extraction that can be used to analyze the geometric structure of licence plates. Note that the contour of a license plate is relatively simple—first of all, it is piecewise linear; second, it is convex. We propose to generalize 3-dominant point extraction to k-dominant point extraction in order to adapt to the complex geometric structure of the contour of a Drosophila embryo. The complexity of these geometric structures mainly lies in the following two aspects: i) the contour of a Drosophila embryo may be concave and ii) the contour of two Drosophila embryos that touch to each other may be concave (see Fig. 1). The proposed method is able to segment embryos touching to each other. Note that many methods on contour extraction, including an active contour model, can not segment two objects that touch to each other. The proposed geometric method can also be applied to other tasks relevant to contour extraction.

## Methods

### 3-dominant point extraction

Given a set of points, such as a connected component of edge pixels, Li et al. [12] proposed a recursive method to extract three dominant points *v*_1_,*v*_2_ and *v*_3_. The first two points (*v*_1_ and *v*_2_) maximize the Euclidean distance of an arbitrary pair of points in *C*, and the third point *v*_3_ maximizes the sum of distances between a *p*∈*C* and *v*_*i*_,*i*=1,2, i.e.,

1 $$  \max_{p\in C} \left(\left\| p-v_{1} \right\| + \left\| p - v_{2} \right\|\right).  $$

Given a point and a line segment *v*_1_ *v*_2_, we call ∥*p*−*v*_1_∥+∥*p*−*v*_2_∥ a *point-line-segment* distance to distinguish the common *point-line* distance that is defined by the distance between *p* and its vertical intersection with a line passing *v*_1_ and *v*_2_.

Based on *v*_1_,*v*_2_, and *v*_3_, the piecewise linearity of *C* is then verified, i.e., where each point *p*∈*C* falls on either the line segment *v*_1_ *v*_3_ or *v*_2_ *v*_3_. If yes, the recursion is stop. Otherwise, *C* is partitioned into two subsets, and the above 3-dominant point extraction method is then applied to the two subsets recursively.

### K-dominant point extraction: basic concepts

In this section, we first generalize the approach for locating the 3rd dominant point, given two dominant points, from a set of unordered points *P* to a formula for locating the *i*-th dominant point, given *i* number of dominant points, from *P*. Then, we propose a simple method to insert a new dominant point into a sequence of geometrically-ordered dominant points so that dominant points are ordered geometrically. Last, we proposed a solution to address the challenge of concave polygons.

The basic idea of k-dominant point extraction is to iteratively insert a new dominant point into a set of *k*−1 dominant points that have been found, under certain geometric constraint. For the convenience of illustration, we now introduce several basic concepts. First of all, the *k*−1 dominant points are expected to be a geometric sequence that is consistent with a given set of 2D points.

Denote 〈*r*(1),…*r*(*k*)〉 is a permutation of 1,…,*k*. A *geometric sequence* of *k*−1 dominant points is denoted as *S*_*k*−1_=〈*v*_*r*(1)_,*v*_*r*(2)_,…,*v*_*r*(*k*−1)_〉. A valid geometric sequence is expected to be a polyline, i.e., *v*_*r*(1)_ *v*_*r*(2)_ is the first line segment passing a subset of points, and *v*_*r*(2)_ *v*_*r*(3)_ is the second line segment passing a subset of points, etc. The initial geometric sequence contains two dominant points, ideally representing a line segment (also called *1-piece polyline*).

A *closed* tag is introduced with respect to a consecutive pair of dominant points (*v*_*r*(*i*)_,*v*_*r*(*i*+1)_) in a geometric sequence with the motivation of speeding up the insertion of a new dominant point. A consecutive pair with (*v*_*r*(*i*)_,*v*_*r*(*i*+1)_) a *closed* tag indicates that a new dominant point is not allowed to be inserted between *v*_*r*(*i*)_ and *v*_*r*(*i*+1)_ in the associated geometric sequence.

*Insertability* is introduced with respect to a new dominant point in order to tell whether the new dominant point is allowed to insert to a geometric sequence of dominant points. Insertability of a point is essentially introduced as a condition to stop the “global” search of dominant points. Imagine that we have a set of points forming a rectangle. After we find out four dominant points associated with the four vertices of a rectangle, the insertability of the fifth dominant point is expected to be NO in order to avoid inserting a non-vertex point into the sequence. Given a geometric sequence *S*=〈*v*_1_,*v*_2_,…,*v*_*k*−1_〉 and a point *v*_*k*_, the point *v*_*k*_ is called (*S*,*ε*)*-insertable*if the point-line-segment distance between *v*_*k*_ to every pair (*v*_*i*_,*v*_*i*+1_) is less than or equal to (1+*ε*) times the length of the line segment *v*_*i*_ *v*_*i*+1_, i.e.,

2 $$  \left\|v_{k} -v_{i}\right\| + \left\|v_{k} - v_{i+1}\right\| \le (1+\epsilon) \left\|v_{i}-v_{i+1}\right\|, \quad \forall i,  $$

where *ε* is a parameter to tolerate the distortion of a straight line. *ε* is not a sensitive parameter, and it can be set from 0.01 to 0.05. In this paper, we fix it to be 0.02. Thus, we sometime simply call a point *v*_*k*_ *S*-insertable, or just insertable.

### Initialization

Similar to 3-dominant point extraction, k-dominant point extraction starts from locating two points *v*_1_ and *v*_2_, given a point set, such that their Euclidean distance is a maximal distance among distances of all pairs of points in *P*, i.e.,

3 $$  \left(v_{1}, v_{2}\right) = \text{argmax}_{p1 \in P, p2 \in P} \left\| p1 - p2\right\|.  $$

The closed tags for *v*_1_ and *v*_2_ are both initialized as 0 (i.e., false).

### Searching a new dominant point

Given a set of points *P* and *k*−1 dominant points *v*_1_,…,*v*_*k*−1_, *k*>2, we propose the following formula to search the *k*-th dominant point:

4 $$  \max_{p\in P} \sum^{k-1}_{i=1} \left\| p-v_{i} \right\|.  $$

For *k*=3, we have an initial geometry sequence *S*_2_=〈*v*_1_,*v*_2_〉. Based on Eq. 2, we can test the insertability of the new dominant point *v*_3_. For *k*>3, we can assume that a geometry sequence *S*_*k*−1_ has been iteratively built, as described in the following section.

It is worth noting that the time complexity of searching a new dominant point depends on the point set *P*, i.e., *Θ*(|*P*|). However, testing the insertability of the new dominant point depends on the geometry sequence *S* only, i.e., in the cost of *Θ*(|*S*|). With closed tags, the computational cost can be further reduced.

### Upon an insertable dominant point: growing *S*_*k*−1_

Given an *S*-insertable dominant point, we will insert the point into an open “slot” of the geometric sequence *S*. Given a sequence *S*_*k*−1_ of *k*−1 dominant points with a geometric order, i.e., *S*_*k*−1_=〈*v*_*r*(1)_,*v*_*r*(2)_,…,*v*_*r*(*k*−1)_〉, each open pair (*v*_*r*(*i*)_,*v*_*r*(*i*+1)_) of *S* offers a space for an insertable *v*_*k*_ to insert, as follows:

5 $$  S_{k-1,i} = \left\langle v_{r(1)}, \ldots, v_{r(i)}, \Box, v_{r(i+1)}, \ldots, v_{r(k-1)} \right\rangle,  $$

where □ indicates a possible space where *v*_*k*_ may be inserted. Here the notation *S*_*k*−1,*i*_ represents an *abstract* geometric sequence that contains a placeholder □ between the pair (*v*_*i*_,*v*_*i*+1_). Furthermore, we denote 〈*S*_*k*−1,*i*_,*v*_*k*_〉 a *concrete* geometric sequence by replacing the placeholder □ in the abstract sequence *S*_*k*−1,*i*_ by *v*_*k*_.

For convenience, we introduce *r*(*k*)=*r*(1) and augment the sequence 〈*v*_*r*(1)_,*v*_*r*(2)_,…, *v*_*r*(*k*−1)_〉 to 〈*v*_*r*(1)_,*v*_*r*(2)_,…, *v*_*r*(*k*−1)_,*v*_*r*(*k*)_〉.

We propose two criteria to measure the confidence of a sequence of dominant points *S*_*k*_=〈*v*_*r*(1)_,*v*_*r*(2)_,…,*v*_*r*(*k*)_〉. The first one is:

6 $$  conf(S_{k}) = \left| \left\{ p \in C: p \in v_{r(i)}v_{r(i+1)} \right\}\right|,  $$

where ∥·∥ denotes the cardinality of a set.

The second criterion is based on overall length of the polyline formed by the sequence *S*_*k*_, i.e.,

7 $$  conf(S_{k}) = \sum^{k}_{i=1} \left\|v_{r(i)}-v_{r(i+1)}\right\|.  $$

The second criterion can be applied if *C* forms a simple curve (no self intersection). It is also easy to see that the computational cost of the second type of confidence is much lower than the first type.

By maximizing the confidence of each derived sequence, we can decide the optimal insertion of a new dominant point in order to maintain the geometric order.

**Fig. 2:**
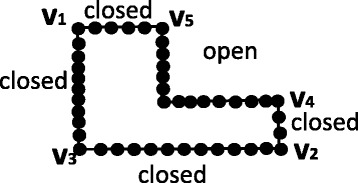
Illutration of open and closed tags. Open and closed tages on a consecutive pair of dominant points in a geometric sequence of dominant points

Figure 2 illustrates an example of assigning a geometric order of *k* dominant points. Given the first dominant points *v*_1_ and *v*_2_. Without lose of generality, we start from the geometric sequence 〈*v*_1_,*v*_2_〉. After *v*_3_ is computed by Eq. 4, there are two possible options to insert *v*_3_: i) 〈*v*_1_,□,*v*_2_〉 and 〈*v*_1_,*v*_2_,□〉. The first option brings us the sequence 〈*v*_1_,*v*_3_,*v*_2_〉, where closed tag assignment is (1,1,0), meaning that *v*_1_ *v*_3_ and *v*_3_ *v*_2_ are closed, and *v*_2_ *v*_1_ is open. The second option brings us the sequence 〈*v*_1_,*v*_2_,*v*_3_〉, where closed tag assignment is (0,1,1), meaning that *v*_1_ *v*_2_ is open, and *v*_2_ *v*_3_,*v*_3_ *v*_1_ are both closed. Based on the confidence measure, these two options are both optimal. Without lose of generality, we choose the first option for following illustration. Consecutively, the proposed method grows the geometric sequence as follows: i) 〈*v*_1_,*v*_3_,*v*_2_〉 with closed tags (1,1,0); ii) 〈*v*_1_,*v*_3_,*v*_2_,*v*_4_〉 with closed tags (1,1,1,0); iii) 〈*v*_1_,*v*_3_,*v*_2_,*v*_4_,*v*_5_〉 with closed tags (1,1,1,0,1).

### Upon a non-insertable dominant point: reduce *P*

If a new dominant point *v*_*i*_ computed by Eq. 4 is non-insertable, we will stop growing the geometric sequence and start to reduce the input point set *P*. The basic idea of reducing *P* is to remove all points that lie in one of closed line segments such that we obtain a subset of points that have a simpler topology. A point remained in *P* must be associated with a certain open pair of dominant points. So the above method can be recursively applied to each subset of points, and in turn each output (a sequence of dominant points from a subset of points can be correctly inserted into a higher level output). Simply speaking, a non-insertable dominant point activates a divide-and-conquer strategy that can handle the concavity of a data set shaped by a concave polygon.

**Fig. 3:**
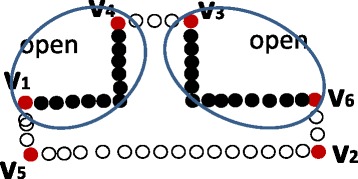
Reduction of a point set. Reducing the input point set *P* if a new dominant point (that is some point in one of four closed line segments) is not insertable. A red dot represents an “old” dominant point, a circle represents a point removed from *P*, and a black dot represents a point in $\bar {P}$ (point set after reduction)

Figure 3 illustrates the basic idea on how to reduce *P* when a new dominant point is tested to be non-insertable. After reduction, there are two subsets of points since there are two open pairs of dominant points in the geometric sequence. K-dominant point extraction method is then recursively applied to these two point sets, respectively.

Algorithm 1 summarizes the procedure of testing the closedness of a pair of dominant points (*v,w*), given a point set *P*. Note that if closedness is true, *P* will be updated by removing all points near the line segment *vw*. The time complexity of the algorithm is dominated by Step 5 (sorting) that is *O*(|*P*| log(|*P*|)). Algorithm 2 summarizes the recursive implementation for k-dominant point extraction. The time complexity of the algorithm is dominated by two non-recursive steps: i) Step 4 (the initialization of *S*) that is *O*(|*P*|^2^), and ii) Step 13 (closedness) that is *O*(|*P*| log(|*P*|), in addition to the recursive step, i.e., Step 16. In many cases, the concavity is not very complicated and the depth of recursion won’t be over 2. Therefore, the overall time complexity of the Algorithm is *O*(|*P*|^2^(1+ log(|*P*|)^2^)) with an assumption that the number of vertices of a polygon is a constant and points in *P* can be uniformly projected to *S*.





### Limitation of max-sum-distance measure

**Fig. 4:**
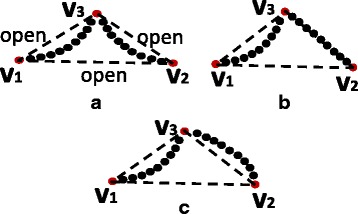
Different scenarios on dominant points and point sets. **a** A scenario where the fourth dominant point is non-insertible, and the point set is non-reducible with respect to *v*_*i*_,*i*=1,…,3; **b** A scenario where the fourth dominant point is non-insertible, and the point set is reducible with respect to *v*_*i*_,*i*=1,…,3; **c** A scenario where the fourth dominant point is insertible with respect to *v*_*i*_,*i*=1,…,3, and the algorithm will continue to locate the fifth dominant point

Figure 4 shows three scenarios of point sets after the first three dominant points have been located. Figure 4 a shows a scenario where the fourth dominant point is non-insertible with respect to the sequence 〈*v*_1_,*v*_2_,*v*_3_〉, however a point set is non-reducible. More specifically, given a point set (a number of black dots) as illustrated in the figure, assume that the first three dominant points have already been located according to the proposed kdp method. Since the max function in Eq. 4 is a convex function, the fourth dominant point is expected to be near to the boundary of the convex hull of the point set (which is equivalent to the triangle with vertices *v*_1_,*v*_2_ and *v*_3_). In other words, the four dominant point must be a point nearest to either *v*_1_,*v*_2_ or *v*_3_. Based on the definition of “insertible”, the fourth point is non-insertible with respect to the geomtric sequence *v*_1_,*v*_2_ and *v*_3_. According to the above algorithm, we now stop growing the sequence, and start to reduce the given point set *P*. However, it turns out that *P* is not reducible. This scenario shows a limitation of using max-sum-distance in computing k-dominant points. Note that this scenario is a representative formuation of two objects who have smooth boundaries and are touching to each other.

Figure 4 b shows a scenario where the fourth dominant point is non-insertible and the point set is reducible with respect to *v*_*i*_,*i*=1,…,3. The fourth dominant point is expected to be a point on the line segment *v*_2_ *v*_3_. Precisely speaking, the fourth dominant point is the point nearest to *v*_2_ as ∥*v*_2_−*v*_1_∥>∥*v*_3_−*v*_1_∥. Note that for any point *p* on the line segement, the sum of distances *v*_2_ *v*_3_, ∥*p*−*v*_2_∥+∥*p*−*v*_3_∥ is a constant. If ∥*v*_2_−*v*_1_∥=∥*v*_3_−*v*_1_∥, the fourth dominant point can be an arbitray point on the line segment *v*_2_ *v*_3_. Unlike the scenario Fig. 4 a where all pairs of dominant points are open, the point set in Fig. 4 b is reducible because (*v*_2_,*v*_3_) forms a closed pair.

Figure 4 c shows a scenario where the fourth dominant point is insertible. Therefore, the algorithm will continue to locate the fifth dominant point. Reducibility is thus not applicable in this scenario.

**Fig. 5:**
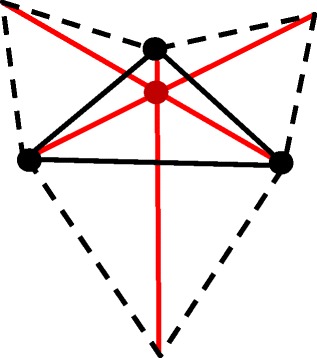
The Fermat point. Given three points (the black dots) in plane, the Fermat point (the red dot) is the point in the plane that minimizes the sum of distances from itself to the three points. The Fermat point can be computed analytically

The rationale of introducing a min-sum-distance can also be found by a Fermat point and its generalization called a geometric median. Given three points in a plane, the *Fermat point* is the point in the plane that minimizes the sum of distances from itself to the three points. The Fermat point can be computed analytically, as illustrated in Fig. 5. Specifically, we first construct an equilateral triangle (in dash lines) for each edge of the given triangle (in solid lines), and then connect the outmost vertex of each equilateral triangle to a given vertex outside the equilateral triangle by a red line. The Fermat point must be the intersection of three red lines. Given *m* points in a plane, the *geometric median* is the point in the plane that minimizes the sum of distances from itself to the *m* points.

A subtle difference between computing a geometric median and computing a dominant point under the min-sum-distance scheme is that the search space in the former problem is a continous and infinite plane, while the search space in the latter problem is a discrete and finite point set. For convenience, we call the problem of computing a dominant point under a min-sum-distance scheme *discrete geometric median*.

### Max/min-sum-distance measure

The three scenarios illustrated in Fig. 4 show that a max-sum-distance scheme is not able to handle various structures of a point set to extract dominant points. A min-sum-distance scheme is expected to be integrated with a max-sum-distance scheme adaptively. In other words, we have to compare both schemes, a max-sum-distance and a min-sum-distance, and select a better scheme under some criterion to extract the next dominant point during the stage of growing the geometric sequence of dominant points. For convenience, we call the problem as *max/min-sum-distance measure problem*.

To solve the max/min-sum-distance problem, we propose a balance-oriented criterion to select an optimal measure between max-sum-distance and min-sum-distance measure as follows: *balance the distance from a new dominant point to its two neighboring dominant points in the new geometric sequence.* Specifically, given a point set *P* and a geometric sequence of dominant points *S*_*k*−1_=〈*v*_1_,…,*v*_*k*−1_〉, we have two candidates of a new dominant point: i) $v^{\max }_{k}$ according to the max-sum-distance measure, and ii) $v^{\min }_{k}$ according to the min-sum-distance measure. For each candidate, $v^{\max }_{k}$ or $v^{\min }_{k}$, we first compute the optimal location to insert by maximizing the confidence of a derived sequence, i.e.,

$$\begin{array}{@{}rcl@{}} i^{*,\max} & = &\text{argmax}_{i} conf\left(\left\langle S_{k-1,i}, v^{\max}_{k} \right\rangle\right) \\ i^{*,\min} & = &\text{argmax}_{i} conf\left(\left\langle S_{k-1,i}, v^{\min}_{k} \right\rangle\right) \\ \end{array} $$

We then compute a distance ratio with respect to $v^{\max }_{k}$, $v^{\min }_{k}$ and their neighboring dominant points as follows:

$$\begin{array}{@{}rcl@{}} r^{\max} & = & \frac{\min\left(\left\|v^{\max}_{k} - v_{i^{*,\max}}\right\|, \left\|v^{\max}_{k} - v_{i^{*,\max}+1}\right\|\right)}{\max\left(\left\|v^{\max}_{k} - v_{i^{*,\max}}\right\|, \left\|v^{\max}_{k} - v_{i^{*,\max}+1}\right\|\right)} \\ r^{\min} & = & \frac{\min\left(\left\|v^{\min}_{k} - v_{i^{*,\min}}\right\|, \left\|v^{\min}_{k} - v_{i^{*,\min}+1}\right\|\right)}{\max\left(\left\|v^{\min}_{k} - v_{i^{*,\min}}\right\|, \left\|v^{\min}_{k} - v_{i^{*,\min}+1}\right\|\right)} \\ \end{array} $$

Note that both *r* ^max^ and *r* ^min^ range from 0 to 1. A larger value indicates more balanced distance from a candidate to its optimal neighbors. So, if *r* ^max^≥*r* ^min^, the candidate $v^{\max }_{k}$ is selected. Otherwise, $v^{\min }_{k}$ is selected.

It is intuitive that dominant points selected by a balance-oriented criterion have a better description of the global structure of a point set, and thus they can provide more reliable estimation of geometric properties of a point set, such as curvature. In contrast, if a dominant point has an imbalanced distance ratio to its two neighbors, the estimation of a geometric property at this dominant point will be very sensitive to the localiation error of itself and its neighbors.

It is easy to see that the balanced-oriented criterion on distance measure can address the above-mentioned three scenarios very well. It is also not difficult to show that the max/min-sum-distance measure can be consistently selected if a point set forms a convex polygonal shape.

### Limitation of max/min-sum-distance measure

**Fig. 6:**
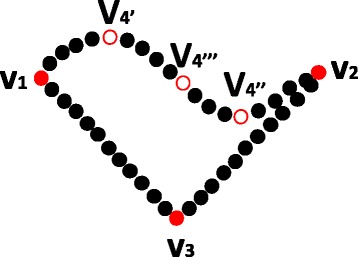
A scenario where a point set contains an inflection point $v_{4^{\prime \prime \prime }}$. $v_{4^{\prime }}$ and $v_{4^{\prime \prime }}$ are two candidates for the 4th dominant point based on max/min-sum-distance. However, the inflection point $v_{4^{\prime \prime \prime }}$ has a more balanced distance ratio to its neighboring dominant points *v*_1_ and *v*_2_ than $v_{4^{\prime }}$ and $v_{4^{\prime \prime }}$

Figure 6 shows a scenario where a point set contains an inflection point $v_{4^{\prime \prime \prime }}$. Since an inflection point is neither a local maximum or a local minimum, the max/min-sum-distance measure fails to extract such a point as a dominant point at an “early” round. (It is possible that an inflection point can be eventually extracted after a few more rounds.) More specifically, three dominant points *v*_1_,*v*_2_ and *v*_3_ are first extracted without any problem. Based on max/min-sum-distance measure, there are two candidates for the 4th dominant point: i) $v_{4^{\prime }}$ according to max-sum-distance measure, and ii) $v_{4^{\prime \prime }}$ according to min-sum-distance measure. However, the inflection point $v_{4^{\prime \prime \prime }}$ has a more balanced distance ratio to its neighboring dominant points *v*_1_ and *v*_2_ than $v_{4^{\prime }}$ and $v_{4^{\prime \prime }}$. Thus, a inflection point, intuitively, can have a better description of the global structure of a point set, say, a sequence of dominant points that can response to inflection points can cover a larger number of points in a given point set than an equal-length sequence of dominant points that cann’t response to inflection points.

### Max/min/median-sum-distance measure

We now generalize the max/min-sum-distance measure to a max/min/median-sum-distance measure. We follow a balance-oriented criterion to select an optimal measure among three measures: i) max-sum-distance, ii) min-sum-distance measure, and iii) median-sum-distance as follows: *balance the distance from a new dominant point to its two neighboring dominant points in the new geometric sequence.* Specifically, given a point set *P* and a geometric sequence of dominant points *S*_*k*−1_=〈*v*_1_,…,*v*_*k*−1_〉, we have two candidates of a new dominant point: i) $v^{\max }_{k}$ according to the max-sum-distance measure, ii) $v^{\min }_{k}$ according to the min-sum-distance measure, and iii) $v^{\text {mdn}}_{k}$ according to the median-sum-distance measure. For each candidate, $v^{\max }_{k}$, $v^{\min }_{k}$ or $v^{\text {mdn}}_{k}$, we first compute the optimal location to insert by maximizing the confidence of a derived sequence, i.e.,

$$\begin{array}{@{}rcl@{}} i^{*,\max} & = &\text{argmax}_{i} conf\left(\left\langle S_{k-1,i}, v^{\max}_{k} \right\rangle\right) \\ i^{*,\min} & = &\text{argmax}_{i} conf\left(\left\langle S_{k-1,i}, v^{\min}_{k} \right\rangle\right) \\ i^{*,\text{mdn}} & = &\text{argmax}_{i} conf\left(\left\langle S_{k-1,i}, v^{\text{mdn}}_{k} \right\rangle\right) \\ \end{array} $$

We then compute a distance ratio with respect to $v^{\max }_{k}$, $v^{\min }_{k}$, $v^{\text {mdn}}_{k}$, and their neighboring dominant points as follows:

$$\begin{array}{@{}rcl@{}} r^{\max} & = & \frac{\min\left(\left\|v^{\max}_{k} - v_{i^{*,\max}}\right\|, \left\|v^{\max}_{k} - v_{i^{*,\max}+1}\right\|\right)}{\max\left(\left\|v^{\max}_{k} - v_{i^{*,\max}}\right\|, \left\|v^{\max}_{k} - v_{i^{*,\max}+1}\right\|\right)} \\ r^{\min} & = & \frac{\min\left(\left\|v^{\min}_{k} - v_{i^{*,\min}}\right\|, \left\|v^{\min}_{k} - v_{i^{*,\min}+1}\right\|\right)}{\max\left(\left\|v^{\min}_{k} - v_{i^{*,\min}}\right\|, \left\|v^{\min}_{k} - v_{i^{*,\min}+1}\right\|\right)} \\ r^{\text{mdn}} & = & \frac{{\min}\left(\left\|v^{\text{mdn}}_{k} - v_{i^{*,{\text{mdn}}}}\right\|, \left\|v^{\text{mdn}}_{k} - v_{i^{*,{\text{mdn}}}+1}\right\|\right)}{\max\left(\left\|v^{\text{mdn}}_{k} - v_{i^{*,{\text{mdn}}}}\right\|, \left\|v^{\text{mdn}}_{k} - v_{i^{*,{\text{mdn}}}+1}\right\|\right)} \\ \end{array} $$

Note that *r* ^max^,*r* ^min^ and *r* ^mdn^ range from 0 to 1. The largest value indicates the most balanced distance from a candidate to its optimal neighbors. For example, if *r* ^max^ is the largest value, the candidate $v^{\max }_{k}$ will be selected.

### Sequence subdivision

**Fig. 7:**
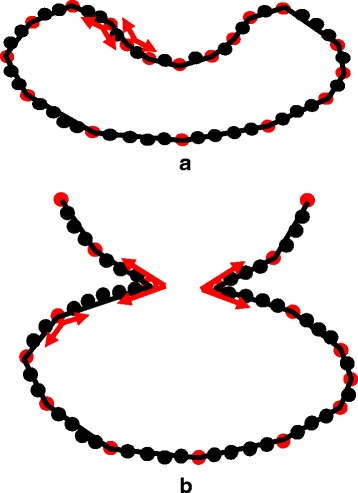
A concave-shape embryo vs. touching embryos. **a** A scenario where the contour of a Drosophila is a bean shape that contains two inflection points. The angles of two inflection points are obtuse. **b** A scenario where the two hollow dots represent the two intersections between the contours of two touching Drosophila embryos, and their angles are acute

We propose a sequence subdivision method for the segmentation of touching embryos. The basic idea of our method is based on the detection of nonsmooth dominant points from a given geometric sequence. To build up an intuition, let us start from Fig. 7 that shows a comparison between a scenario of a concave-shape embryo and a scenario of touching embryos. Given a dominant point *v*_*i*_, denote *v*_*i*−1_ *v*_*i*_=*v*_*i*−1_−*v*_*i*_ and *v*_*i*+1_ *v*_*i*_=*v*_*i*+1_−*v*_*i*_ are two directional vectors centered at *v*_*i*_. The cross angle between these two vectors that can be computed by $\text {acos}\frac {v_{i-1}v_{i}}{\|v_{i-1}v_{i}\|}\frac {v_{i+1}v_{i}}{\|v_{i+1}v_{i}\|}$ can be used to tell whether a dominant point is smooth or not. For simplicity, we call the cross angle of the two directional vectors centered at *v*_*i*_ *the angle* of *v*_*i*_. Figure 7 a shows a scenario where the two hollow dots represent the two intersections between the contours of two touching Drosophila embryos, and their angles are acute. Figure 7 b shows a scenario where the contour of a Drosophila is a bean shape that contains two inflection points. The angles of two inflection points are obtuse, i.e., similar to the cross angle of other dominant points given a smooth contour.

**Fig. 8:**
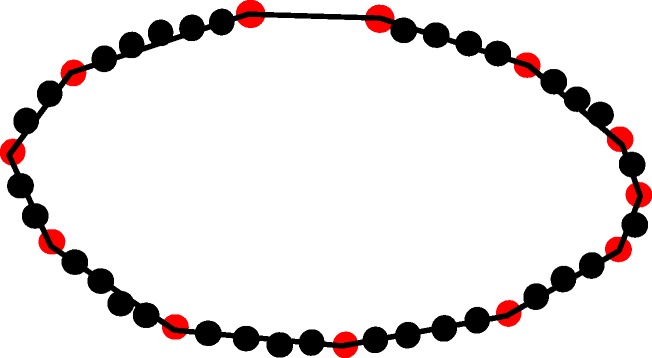
The longest subsequence output by Algorithm 3. To enhance the intuition, the subsequence is drawn over the corresponding subset of input points that are, however, not involved in Algorithm 3

Figure 8 shows the longest subsequence output by the Algorithm 3 being applied to the geometric sequence presented in Fig. 1 b.

## Results

We tested the proposed method on BDGP (Berkley Drosophila Genome Project) [1]. BDGP images are available at the following public webpage: http://www.fruitfly.org/insituimages/insitu_images/.

**Fig. 9:**
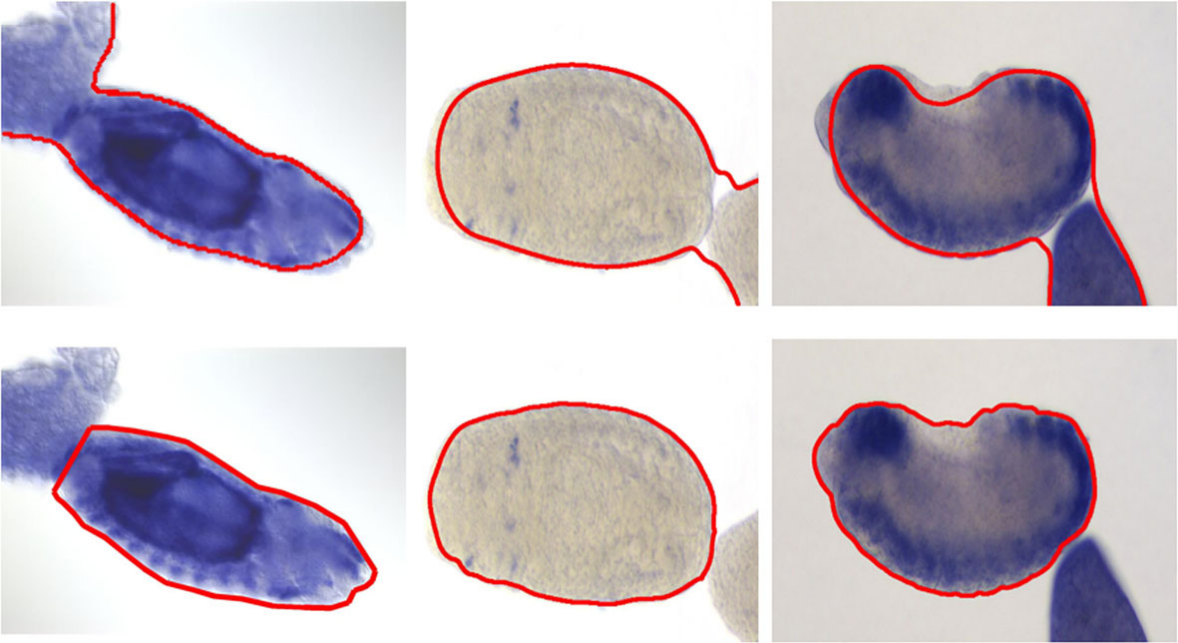
A comparison between Li’s method [4] (the first row) and the proposed method (the second row)

**Fig. 10:**
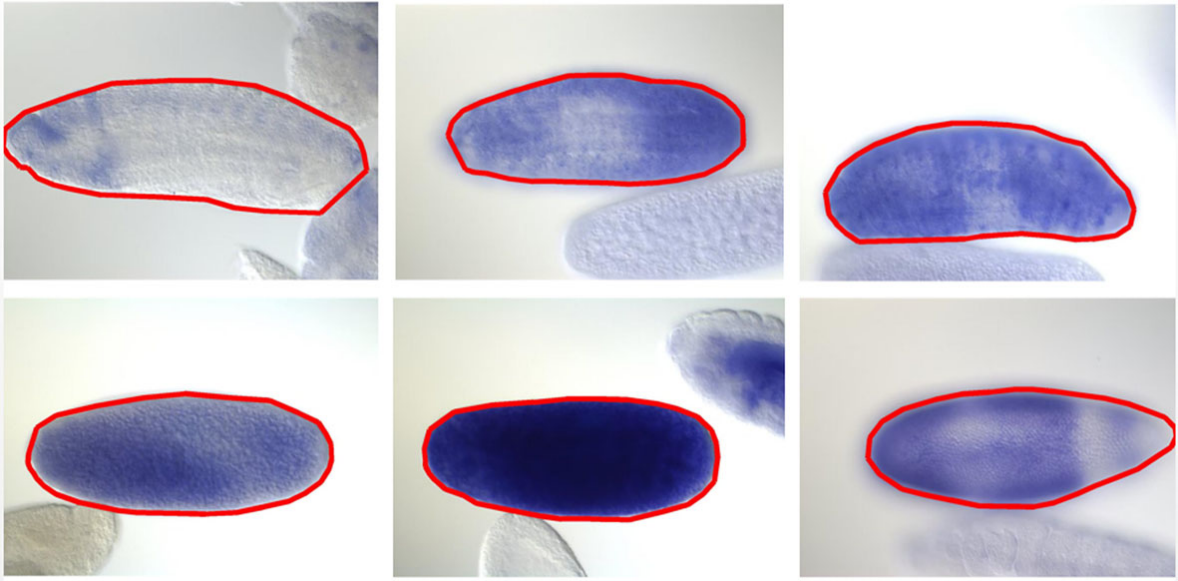
More positive examples of the proposed algorithm on images of touching Drosophila embryos



We first give a visual evaluation on the proposed method. Figure 9 shows a comparison between Li’s method [4] and the proposed method on BDGP images of touching Drosophila embryos. We can observe that Li’s method fails in all three cases, while the proposed method works well. Figure 10 shows more positive results of the proposed method.

Next, we present a quantitative evaluation of different combinations of the parts of the proposed method in terms of detection rates. Given an image, the result is defined as a *successful detection* if the output by Algorithm 2 has larger than 90% overlapping region with the ground truth. The dataset contains 2000 images of BDGP Drosophila embryos. Table 1 shows the quantitative results, including the comparison of two existing methods on contour extraction of Drosophila embryos: i) Li and Kambhamettu’s method [3] that consists an initialization based on a quadratic curve model, and a refinement based on an active contour model; ii) Li’s method [4] that can detect and restore deficiencies and faults of primal sketch tokens occurring when a targeting object is surrounded by a complex background.

**Table 1:** Quantitative results

Method	Detection rate (%)
Li and Kambhamettu [3]	90
Li [4]	92
Proposed	94

## Discussion

As we mentioned in the introduction, the proposed framework can be applied to other applications of contour extraction. The main contribution of the proposed framework is k-dominant point extraction based on a specific distance measure. There is a trade off among the three types of distance measures: max-sum, max/min-sum, and max/min/median-sum. The last one is the most sophisticate one, i.e., it can deal with concave contours, and touching scenarios of targeting objects, while the first one is the most efficient one in computation. Therefore, max-sum distance may be used in some circumstance, e.g., contours are convex. K-dominant point extraction may also be applied to more general applications beyond contour extraction, such as data clustering. In other words, the data that k-dominant point extraction is applied can have arbitrary dimensions rather than two.

## Conclusions

We have proposed a geometric method for contour extraction of Drosophila embryos. Experiment results show the effectiveness of the proposed method, typically in segmenting two touching embryos in an image. The results also show the superiority of the proposed method over two previous methods. The proposed method advances the theory of control point detection by generalizing 3-dominant points to k-dominant points. The generalization includes strategies to deal with concave shapes, thus the proposed method can be applied to a wide range of applications relevant to contour extraction.
